# High-resolution aging niche of human adipose tissues

**DOI:** 10.1038/s41392-023-01315-9

**Published:** 2023-03-15

**Authors:** Wenyan Zhou, Junxin Lin, Yan Xie, Xueqing Hu, Xudong Yao, Yuemin Ou, Hongwei Wu, Yiyang Yan, Xiumao Li, Jianan Lou, Aaron Trent Irving, James Q. Wang, Hongwei Ouyang

**Affiliations:** 1 Dr. Li Dak Sum & Yip Yio Chin Center for Stem Cells and Regenerative Medicine, and Department of Orthopedic Surgery of the Second Affiliated Hospital, Zhejiang University School of Medicine, Hangzhou, 310058 China; 2 Department of Sports Medicine, Zhejiang University School of Medicine, Hangzhou, 310058 China; 3 Zhejiang University-University of Edinburgh Institute, Zhejiang University School of Medicine, and Key Laboratory of Tissue Engineering and Regenerative Medicine of Zhejiang Province, Zhejiang University School of Medicine, Hangzhou, 310058 China; 4 Tissue Organ Bank & Tissue Engineering Centre, General Hospital of Ningxia Medical University, Ningxia, 750003 China; 5 Department of Plastic Surgery, The Second Affiliated Hospital, Zhejiang University School of Medicine, Hangzhou, 310052 China; 6 The Fourth Affiliated Hospital, Zhejiang University School of Medicine, Yiwu, 322000 China; 7 Department of Orthopedics, The Second Affiliated Hospital, Zhejiang University School of Medicine, Hangzhou, 310009 China; 8 China Orthopedic Regenerative Medicine Group (CORMed), Hangzhou, 310058 China

**Keywords:** Senescence, Ageing

**Dear Editor**,

Age-dependent adipose tissue malfunction raises the risk of diseases like diabetes, cardiovascular disease, and even cancer by contributing to metabolic decline, heterotopic fat storage, and chronic systemic inflammation.^1^ Understanding adipose tissue aging requires in-depth knowledge of the cellular and molecular properties of various adipose tissue cell types. Although the heterogeneity of the cell population during mouse aging has been studied,^2^ little is known about the cellular and molecular basis of human adipose tissues aging.

Our single-cell RNA sequencing (scRNA-seq) analysis on stromal vascular fraction cells of subcutaneous adipose tissues (SAT) derived from young and old humans identified 12 cell populations, including adipose progenitor cells (APC, *PDGFRA*^*+*^), immune cells (IC, *PTPRC*^*+*^), vascular endothelial cells (VEC, *PECAM1*^*+*^), and smooth muscle cells (SMC, *ACTA2*^*+*^) (Fig. 1a, b and supplementary Fig. S1, supplementary Table S1). Seven APC populations contributed to the majority of the stromal vascular fraction cells of SAT. APC3 was recognized as “interstitial progenitors” for expressing the highest level of stem cell markers (*CD55, PI16, SEMA3C, DPP4*) when compared to other progenitors (APC1, APC2, APC4, APC6 and APC7), which expressed higher level of early adipogenic markers (*FABP4, APOE, GGT5, DEPP1*) (supplementary Fig. S2a). Gene Ontology analysis further revealed one preadipocyte population (APC4), two extracellular matrix (ECM)-secretory population (APC3 and APC6), and two inflammatory APC population (APC5 and APC7) (supplementary Fig. S2b, c and supplementary Table S2).

**Fig. 1:**
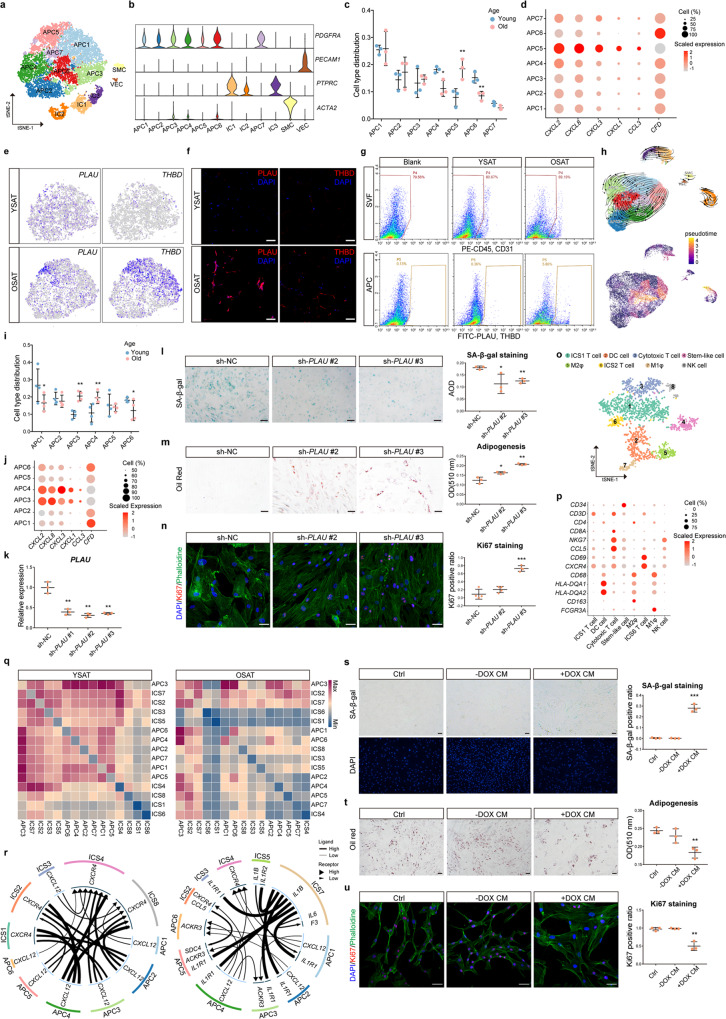
Aging of human adipose tissues is accompanied by the appearance of PLAU^+^ adipose progenitor cells and dominating role of macrophages. **a** t-Distributed stochastic neighbor (t-SNE) plot shows unsupervised clustering of 13841 single-cell transcriptomes of SVF cells from 3 young individuals and 3 old individuals. **b** Violin plots show the linage marker genes expression level in all cell clusters. **c** Scatter plot shows the changes in the proportion of 7 APC subpopulations during the aging process. Data are presented as the mean ± s.d. of three young and three old individuals. **d** Dot plot shows the expression levels of chemokine activity-related genes and adipogenic-related genes across 7 APC subpopulations of 3 young and 3 old individuals. **e** t-SNE plots show the expression level of *THBD* and *PLAU* in all APC clusters in young and old individuals. **f** Representative images of immunofluorescence staining of PLAU and THBD in human SAT of young and old group. Scale bar, 50 μm. **g** FACS analysis of PLAU^+^, THBD^+^ APC in SAT of young and old group. **h** UMAP visualization of all APC subpopulations. RNA-velocity analysis with velocity field projected onto the UMAP plot of APC subpopulations, arrows show the local average velocity evaluated on a regular grid and indicate the extrapolated future states of cells (top). Cells were annotated by monocle pseudotemporal dynamics (purple to yellow, below). **i**, **j** APC populations of the second cohort. **i** Scatter plot shows the changes in the proportion of 6 APC subpopulations during SAT aging process. Data are presented as the mean ± s.d. of 4 young and 4 old individuals from the second cohort. **j** Dot plot shows the expression levels of chemokine activity-related genes and adipogenic-related gene across 6 APC subpopulations of 4 young and 4 old individuals from the second cohort. **k** qRT-PCR for *PLAU* expression level of human APC after *PLAU* knockdown. Data are presented as the mean ± s.d. of three biological repetitions. **l** Representative images of SA-β-gal staining of human APC upon shRNA-mediated knockdown of *PLAU*. Scale bar, 50 μm. Positive signals were quantized by average optical density (AOD). Data are presented as the mean ± s.d. of 3 fields of each group. **m** Representative images of Oil Red staining shows the adipogenic differentiation capacity of human APC upon shRNA-mediated knockdown of *PLAU*. Scale bar, 50 μm. Data are presented as the mean ± s.d. of 3 cell wells of each group. **n** Representative images of immunofluorescence staining of Ki67 in human APC upon shRNA-mediated knockdown of *PLAU*. Scale bar, 50 μm. Data are presented as the mean ± s.d. of 4 fields of each group. **o** Re-clustering of IC1-IC3 in **a**, **b** identified 8 specific immune cell subpopulations, samples from 3 young individuals and 3 old individuals. **p** Dot plot shows the expression levels of representative cell-type-specific marker genes across all these 8 immune cell subpopulations. **q** Interaction heatmap plots the total number of cell receptor (*y* axis) and ligand (*x* axis) interactions in 3 young donors (left) and 3 old donors (right) derived SAT. The color key from blue to red indicates low to high number of interactions. **r** Circos plots shows the top 20 chemokines mediated ligand-receptor interaction for all APC and immune cell subpopulations (left, young group, *n* = 3; right, old group, *n* = 3). **s**–**u** Primary human APC treated with conditioned media from DOX-induced senescent macrophages. **s** Representative images of SA-β-gal staining of conditioned media treated APC. Scale bar, 50 μm. Data are presented as the mean ± s.d. of 3 fields. **t** Representative images of Oil Red staining show the adipocyte differentiation of conditioned media treated APC. Scale bar, 50 μm. Data are presented as the mean ± s.d. of 3 biological replications. **u** Representative images of immunofluorescence staining of Ki67 in conditioned media treated APC. Scale bar, 50 μm. Data are presented as the mean ± s.d. of 3 fields. YSAT, young subcutaneous adipose tissues; OSAT, old subcutaneous adipose tissues; -DOX CM, conditioned medium of macrophages without DOX treatment; +DOX CM, conditioned medium of macrophages treated by DOX. In dot plot (**d**, **j**, **p**), the size of the dot corresponds to the percentage of cells expressing the specific gene in each cluster, and the color encodes the scaled average expression level of feature genes across all cells within a subpopulation. *P < 0.05; **P < 0.01; ***P < 0.001; ***P < 0.001

Interestingly, it was noted that the cell number of inflammatory APC5 increased significantly with aging (Fig. 1c). Analysis of aging-dependent differentially expressed genes showed that the expression level of cell surface markers *PLAU* and *THBD* were significantly increased in aged APC5 (Fig. 1e and supplementary Table S3). Immunofluorescence staining and flow cytometry of human adipose tissues based on *PLAU* and *THBD* verified the accumulation of APC5 in old individuals (Fig. 1f, g). Further examination of the marker genes of APC subpopulations revealed that PLAU^+^ APC5 expressed highest level of chemokine genes (*CXCL2*, *CXCL8*, *CXCL3*, and *CXCL1*), but lower level of functional gene (*CFD*) than those of the other APC subpopulations (Fig. 1d). To trace the origin of PLAU^+^ APC, we performed RNA velocity analysis. The velocity stream suggested a transition from “interstitial progenitors” APC3 through ECM-secretory APC6 and preadipocyte APC4, then to the dysfunctional PLAU^+^ APC5 at the end (Fig. 1h). These findings indicated that human SAT contain a subset of defective inflammatory APC population that accumulates with aging. In scRNA-seq analysis on SAT from another independent cohort of young and aged donors, we also identified inflammatory APC populations (APC3 and APC4) that accumulate with age and exhibit higher levels of *PLAU* and *THBD* expression, further supporting the notion. (supplementary Fig. S3, Fig. 1i, j, supplementary Table S1, and supplementary Table S4).

As one of the biomarkers of APC5, *PLAU* was also predicted as a frailty marker in previous study.^3^ Our western blotting results also verified PLAU protein level was higher in SAT of old individuals (supplementary Fig. S4). Thus, we speculated that *PLAU* might be involved in the progression of APC aging. To test this hypothesis, we firstly measured the expression level of *PLAU* in human primary APC derived from young (16-year-old) and old (69-year-old) individuals. The qRT-PCR results showed that *PLAU* expression level was positively correlated with donor age (supplementary Fig. S5a). Additionally, the adipogenic differentiation capacity of APC from elderly individuals was significantly lower than that of young individuals (supplementary Fig. S5b). Senescence-associated β-galactosidase (SA-β-gal) staining showed that overexpression of *PLAU* (overexpressing efficiency, 178.8% ± 24.2%) promoted senescence of human primary APC (supplementary Fig. S5c, d). Next, we investigated whether down-regulation of *PLAU* could rescue APC aging. Knockdown of *PLAU* (silencing efficiency, 60.6% ± 7.3% for sh-PLAU#1, 69.1% ± 5.1% for sh-PLAU#2, 64.3% ± 2.2% for sh-PLAU#3) in primary APC isolated from old donors (>65 years) significantly alleviated cell senescence, as well as enhanced adipogenic and proliferation potential of aged human primary APC (Fig. 1k–n). Collectively, these results suggested that aberrant expression of *PLAU* promotes APC aging, and may represent a promising therapeutic target for aging related diseases of human adipose tissues.

Previous studies showed that adipose tissue functions are tightly regulated by the crosstalk between APC and immune cells.^4,5^ To investigate whether the crosstalk between APC and immune cells contributes to APC aging, we performed an in-depth analysis on immune cell profiles and their communications with APC. Re-clustering of IC1-IC3 cells identified 8 specific immune cell subpopulations (ICS1-8). ICS1, ICS3, ICS6 showed gene expression signatures of distinct T cell subpopulations (Fig. 1o, p). While ICS6 expressed higher levels of the early T cell activation markers *CD69* and *CXCR4*, ICS1 was distinguished from ICS6 by high expression levels of ribosomal protein-related genes, representing a proliferating cell population (supplementary Table S5). ICS3 was annotated as cytotoxic CD8^+^ T cell for the unique expression patterns of *CD8A*, *NKG7*, *CCL5*. ICS2, ICS5, and ICS7 highly expressed monocyte/macrophage markers *CD68* and antigen presentation-related genes (*HLA-DQA1*, *HLA-DQA2*). ICS5 was identified as M2-like macrophage by the expression of *CD163*, and ICS7 was recognized as M1-like macrophage for high expression level of *FCGR3A* (Fig. 1p). After cell type annotation, we utilized CellPhoneDB and iTALK to perform unbiased ligand-receptor interaction analysis between the ICS and APC. Both algorithms demonstrated that M1-like macrophage (ICS7) maintained a relatively strong interaction with other cell subpopulations (Fig. 1q, r). Particularly, iTALK analysis showed that aging shifts the intercellular chemokine crosstalk from an APC-dominating pattern to an M1-like macrophage-dominating pattern (Fig. 1r), suggesting a role of macrophages in regulating the aging microenvironment of SAT.

To study whether the macrophage-dominating interaction pattern contributes to APC dysfunction, we cultured human primary APC derived from young individuals with conditioned medium of Doxorubicin (DOX)-induced senescent M1 macrophages (supplementary Fig. S6a, b). Conditioned medium of senescent macrophages significantly increased *PLAU* expression level in young human primary APC (supplementary Fig. S6c), as well as elevated the expression level of *CXCL2*, *CXCL3*, *CXCL8*, which are markers of aging-related dysfunctional PLAU^+^ APC5 population (supplementary Fig. S6d). Moreover, conditioned medium treatment promoted senescence of human primary APC (Fig. 1s), impaired their adipogenic potential (Fig. 1t) and proliferation capacity (Fig. 1u). To further confirm the macrophage-dominating interaction pattern, CD11c^+^CD206^-^ M1 macrophages were isolated from SAT of young and old individuals and co-cultured with young APC (supplementary Fig. S7a, b). Results also showed that M1 macrophages of aged SAT significantly elevated the inflammation genes expression of APC (supplementary Fig. S7c). Overall, our findings demonstrated that senescent macrophages play a critical role in promoting APC aging.

In summary, we found that as human adipose tissue aging, the intercellular communication microenvironment shifts from being dominated by APC to being dominated by inflammatory M1 macrophages, resulting in the accumulation of a dysfunctional PLAU^+^ APC population. Although other cell types might also participate in aging process,^2^ our study sheds light on the role of chemokine signaling secreted by senescent macrophages and PLAU^+^ APC as prospective diagnostic basis and therapeutic targets for the prevention of aging-related adipose tissue dysfunction.

## Supplementary information

Supplementary materials

Supplementary Table S1

Supplementary Table S2

Supplementary Table S3

Supplementary Table S4

Supplementary Table S5

Supplementary Table S6

Supplementary Table S7

## Data Availability

The raw sequencing data have been deposited to Genome Sequencing Archive of the National Genomics Data Center (accession: HRA001279). All other data and materials used in this work are available from the lead corresponding author (hwoy@zju.edu.cn) upon request. The code to reproduce the analyses and figures described in this study can be found at: https://github.com/TGXBio/huACA.
