# Supplementary material for: High-resolution aging niche of human adipose tissues
Source: Signal Transduct Target Ther. 2023 Mar 15;8:105. doi: 10.1038/s41392-023-01315-9 (PMC10014933; doi:10.1038/s41392-023-01315-9)
Supplement: Supplementary file 1 — Supplementary materials [file 41392_2023_1315_MOESM1_ESM.docx]

**Supplementary Materials for**

**High-resolution aging niche of human adipose tissues**

Wenyan Zhou^1,2,3 §^, Junxin Lin^1,2,3 §^, Yan Xie^5 §^, Xueqing Hu^6^, Xudong Yao^7^, Yuemin Ou^3^, Hongwei Wu^1,2,3^, Yiyang Yan^1,2,3^, Xiumao Li^8^, Jianan Lou^8^, Aaron Trent Irving^3^, James Q. Wang^3^, Hongwei Ouyang^1,2,3,4^*

^1^ Dr. Li Dak Sum & Yip Yio Chin Center for Stem Cells and Regenerative Medicine, and Department of Orthopedic Surgery of the Second Affiliated Hospital, Zhejiang University School of Medicine, Hangzhou, 310058, China

^2^ Department of Sports Medicine, Zhejiang University School of Medicine, Hangzhou, 310058, China

^3^ Zhejiang University-University of Edinburgh Institute, Zhejiang University School of Medicine, and Key Laboratory of Tissue Engineering and Regenerative Medicine of Zhejiang Province, Zhejiang University School of Medicine, Hangzhou, 310058, China

^4^ China Orthopedic Regenerative Medicine Group (CORMed), Hangzhou, 310058, China

^5^Tissue Organ Bank & Tissue Engineering Centre, General Hospital of Ningxia Medical University, Ningxia, 750003, China

^6^Department of Plastic Surgery, The Second Affiliated Hospital, Zhejiang University School of Medicine, Hangzhou, 310052, China

^7^ The Fourth Affiliated Hospital, Zhejiang University School of Medicine, Yiwu, 322000, China

^8^Department of Orthopedics, The Second Affiliated Hospital, Zhejiang University School of Medicine, Hangzhou, 310009, China

§ These authors contributed equally to this work

* Corresponding author

Correspondence to: hwoy@zju.edu.cn

**This PDF file includes:**

Materials and Methods

Extended Discussion

Supplementary Figure S1 to S7

Supplementary Table S1 to S7

**Other Supplementary Materials for this manuscript include the following:**

Original film of Western blot

Materials and Methods

**Study subjects**

Human adipose tissues were obtained from patients undergoing specific surgical procedure with the approval of the ethics committee of Second Affiliated Hospital, Zhejiang University (Approval number: 2018-037) and General Hospital of Ningxia Medical University (Approval number: KYLL-2020-18), and sampling was performed with the patients’ informed consent. Specifically, man and woman who was younger than 30 or older than 65, had a body mass index between 18.5 kgm^-2^ and 30 kg m^-2^ and required surgery which could harvest subcutaneous adipose tissues were included. Patients with the following situations were excluded: systemic autoimmune disease, not yet controlled liver, kidney, lung, and endocrine system diseases, a history of malignancy, viral hepatitis, HIV, syphilis and other infectious diseases, pregnant or lactating women. For scRNA-seq, the young group included patients between the ages of 16 to 29 years old, the old group included patients between the ages of 68 to 87 years old. The characteristics of included patients were in Supplementary Table S1.

**Stromal vascular fraction (SVF) isolation**

SVF cells were collected as previously described^1^. Briefly, adipose tissues were digested at 37°C using collagenase type I (Gibco). SVF cells were collected every 30 min, and fresh collagenase type I were added to the residual adipose tissues until 95% tissues were digested. Then the SVF cells well treated with red blood cell lysis buffer to remove red blood cells. Cells were resuspended with full culture medium (Low glucose-DMEM (Gibco)+10% FBS (Gibco)), and then freezing medium was added. Finally, cells were placed in programmed cooling box at -80°C for 24 hours, and then stored in liquid nitrogen until analysis.

**Single-cell RNA-seq data processing**

The scRNA-seq experiment was performed by Genergy Biological Technology (Shanghai, China) using the Chromium Single Cell Gene Expression Solution (10x Genomics), following the manufacturer’s protocol^2,3^. The Single-cell RNA-seq libraries were sequenced on the HiSeq X Ten Sequencing System (Illumina). Raw reads were aligned against the GRCh38 reference genome and unique molecular identifiers (UMI) were quantified using the 10x Genomics Inc. software package Cell Ranger (v2.1.0) with default parameters^4^. Cells from individual donors were computationally demultiplexed using the souporcell (v.2.4) algorithm, which identifies genotypic differences between single cells by variant calling aligned read without the need for a reference genotype ^5^. The output of the variant calls was also used to identify potential multiplets^5,6^. Quality control was applied to cells based on the number of UMI counts, total detected genes and proportion of mitochondrial gene counts per cell^2^. Specifically, cells with less than 500 detected genes were filtered, as well as cells with more than 10% mitochondrial gene counts. To further remove potential multiplets, cells with total UMI counts above 20,000 or 30,000 detected genes were filtered out depending on different samples. The characteristics of data quality were in Supplementary Table S6.

**Cell clustering and cell-type identification**

Seurat R package (version 3.2.3) was used to perform downstream analysis based on the filtered UMI matrices^7^. To cluster the cells, individual samples were firstly integrated using SCTransform workflow in Seurat. Specifically, variable genes were identified using the *SelectIntegrationFeatures* function with nfeatures = 1000. Integration anchors across all samples were discovered using the *FindIntegrationAnchors* function command with default parameters. The *IntegrateData* function was run on the anchor set to integrate all samples with default arguments. Dimensionality reduction was performed with *RunPCA.* Then t-stochastic neighboring embedding method (tSNE) dimensionality reduction was carried out and Shared Nearest Neighbour (SNN) graph was constructed using dimensions 1-18 (for samples from first cohort, young and old patient 1 to 3) or 1-15 (for samples from second cohort, young and old patient 4-7), which were determined by elbow plot, as input features. Cell clustering was performed on the integrated assay using a resolution of 0.5. Subclustering of immune cells were performed using dimensions 1-6 (determined by elbow plot) and resolution 0.4. Marker genes for each cluster were determined by using the function *FindConservedMarkers* (min.pct = 0.1, logfc.threshold = 0.25, adjust p value < 0.05)*.* Gene Ontology (GO) enrichment analysis DEGs was performed by clusterProfiler (version 3.14.3)^8^. Representative terms selected from the top ranked GO terms (*P* < 0.05) were displayed. Differences in cell type proportions were calculated using the propeller function, which performs a logit transformation on the matrix of proportions and uses limma framework to fit a linear model for each cell type^9^.

**Identification of Aging-Associated DEGs**

We used the function of *FindMarkers* in Seurat to identify aging-associated differentially expressed genes (DEGs) between the old and young groups for each cell type. The log fold change (LogFC) and adjusted p value of each DEG were calculated by using the non-parametric two-sided Wilcoxon rank-sum test and only those with |avg_logFC| > 0.25 and p_val_adj < 0.05 were considered to be aging-associated DEGs.

**Cell-cell communication analysis between APC and immune cells**

Cell-cell interactions between APC and immune cells was performed based on the scRNA-seq data by using iTALK^10^ and CellphoneDB^11^. For iTALK (v.0.1.0), top 50% highly expressed genes were used for further analyses. The software built-in database containing a total of 2,648 unique ligand-receptor interacting pairs were used to identify significant interactions. The top 20 significant interactions were visualized based on the R package circlize^12^. For CellphoneDB (v.2.0.0) analysis, the raw counts and cell type annotation for each cell were input into CellphoneDB to determine the potential ligand–receptor pairs. Pairs with *P* < 0.05 were selected for further analysis.

**RNA velocity and pseudotime trajectory analysis**

To calculate the RNA velocity of single cells, we used the velocyto (v.0.17.17) to generate a loom file containing spliced and unspliced mRNAs in each sample^13^. Then, we used the python package scVelo (v.0.2.3) to recover the directed transcriptional dynamics by leveraging RNA-splicing information^14^. All scVelo functions were used with default parameters. The velocity vectors were obtained using the *velocity* function. The velocities were projected and visualized using the Seurat UMAP embedding using the *velocity_graph* and *velocity_embedding_stream* function. Pseudotime trajectories were built using the R package Monocle 3 (v.1.0.0)^15^. We converted the Seurat object to a CellDataSet object using the *as.cell_data_set* function. Cells of cluster APC3 were specified as root cells. The heatmap was generated using function *plot_cells*.

**Histology and immunofluorescence staining**

Histology and immunofluorescence staining were performed as previously described^16^. Briefly, tissues were fixed with 4% paraformaldehyde over 24 hours, followed by dehydrated through a series of ethanol washes and then embedded in paraffin for thin sectioning. Immunofluorescence staining was performed as previously described. Paraffin-embedded sections were first deparaffinized in 100% xylene washes and rehydrated in series of graded alcohols (100%, 100%, 90%, 80%, 70%, 50%) and finally washed in distilled water. The sections were treated by heat mediated antigen retrieval with sodium citrate buffer (pH = 6.0) for 30 min. 5% BSA was used to block the sample at room temperature for 30 min. Primary antibodies (anti-Urokinase, Huabio#ET1703-26, 1:100; anti-Thrombomodulin, abcam#ab109189, 1:100) were applied in 1% BSA and incubated at 4°C overnight. Nest day, after a wash with PBS, appropriate fluorescence-labeled secondary antibodies (Goat anti-rabbit IgG H&L (Alexa Fluor® 555), abcam#ab150078, 1:200) were added at room temperature for 2 hours. Finally, the sections were stained with DAPI (Beyotime#C1002,1:6000) for nuclear staining. For Ki67 staining, cells were fixed with 4% paraformaldehyde for 15 min at room temperature, permeabilized with 0.1% Triton X-100 in PBS for 10 min and then blocked with 1% BSA in PBS for 1 hour at room temperature. Then the cells were incubated overnight at 4°C with anti-Ki67 (abcam#ab16667,1:200), followed by a further incubation at room temperature for 1 hour with Goat anti-rabbit IgG H&L (Alexa Fluor® 555) (abcam#ab150078, 1:200). The cytoskeleton was stained with phalloidin (Cytoskeleton#PHDG1).

**Western blotting**

Adipose tissues were treated with RIPA supplemented with protease inhibitors and homogenized using tissue grinder (Shanghai Jingxin). Homogenized samples were centrifuged to remove the lipid layer. The aqueous layer was incubated at 4°C for 30 min followed by re-centrifuging to remove residual lipid. BCA kit (Thermo#23227) was used to perform protein quantification. Protein lysates supplemented with loading buffer were subjected to SDS-PAGE and subsequently electro-transferred to a polyvinylidene fluoride membrane. Broad multicolor pre-stained protein standard (GenScript#M00624) was loaded to approximate sizing of proteins. The membrane was incubated with the indicated primary antibodies (anti-Urokinase, proteintech#17968-1-AP, 1:4000; anti-actin, Affinity#T0022, 1:5000) overnight at 4°C and HRP-conjugated secondary antibodies (anti-rabbit HRP, Jackson#111-035-003, 1:5000; anti-mouse HRP, Jackson#115-035-003, 1:5000) for 1 hour at room temperature. The signals were activated by incubation with enhanced chemiluminescence western blotting detection kit (Beyotime#P0018AS), and visualization was performed using western blot imaging system (LI-COR). Data quantification was performed by Image J.

**Flow cytometry**

Flow cytometry was performed following manufacturer’s protocol. SVF cells were incubated with indicated fluorophore-conjugated antibodies (anti-human CD45-PE, Biolegend#368510, anti-human CD31-PE, eBioscience#12-0311-81) and primary anti-Thrombomodulin, abcam#ab109189, anti-Urokinase, Huabio#ET1703-26 at 4°C for 30 min in the dark. Next, cells were washed with PBS buffer and incubated in fluorescence-labeled secondary antibodies (Goat anti-rabbit IgG H&L (Alexa Fluor® 488). Before analysis, cells were stained with DAPI (Beyotime#C1002,1:6000) for dead cell distinguishing. Flow cytometry was performed on ACAE NovoCyte flow cytometer.

**Cell culture**

*Human primary adipose progenitor cells (APC).* APC were cultured as previously described^17^. The SVF cells were cultured in 37°C, 5% CO_2_ with APC medium consisting Dulbecco’s Modified Eagle Medium (DMEM) supplemented with 1 g/L of glucose (Gibco), 10% FBS (Gibco) and 1% penicillin/streptomycin (Life Technologies).

*Senescence macrophage induction.* Human monocytic cell line THP-1was cultured by RPMI 1640 (Gibco) supplemented with 10% FBS, 1% penicillin/streptomycin. PMA (Beyotime#S1819) was used to induce macrophages differentiation by 48 hours incubation at the concentration of 100 ng/ml, followed by a resting period of 24 hours in medium without PMA^18^. PMA induced macrophages were treated by 800nM Doxorubicin (DOX, Sigma#25316-40-9) for 24 hours to induce senescence.

To generate conditioned medium from senescence macrophage, THP-1 was cultured at 1🞨10^6^ cells/ml. After DOX treatment^19^, cells were rinsed with PBS twice to get rid of the residual DOX and then cultured in full medium (RPMI 1640 (Gibco) supplemented with 10% FBS, 1% penicillin/streptomycin) for another 24 hours, then the supernatant was collected as conditioned medium and stored at -80°C. Human APC were plated at 6000 cells/cm^2^ in APC medium. Before activation, APC were serum starved overnight in APC medium without FBS, then the conditioned medium from senescence macrophages was added for 24 hours.

**Macrophages isolation and co-culture**

Macrophages were isolated from frozen SVF. After thawed, SVF cells were incubated with indicated fluorophore-conjugated antibodies (anti-human CD45-PE, Biolegend#368510, anti-human CD11c-BB515, Biolegend#564491, anti-human CD206-BV605, Biolegend#321139) at 4°C for 30 min in the dark. Next, cells were washed with PBS buffer and CD45^+^CD11c^+^CD206^-^ cells were isolated by BD Influx cell sorter.

Young APC were seeded in 24 well-plate at a density of 1🞨10^4^ per well one day before co-culture. The isolated macrophages were seeded on transwell inserts (Corning, #CLS3413) with 0.4 μm pore size polycarbonate permeable membrane at a density of 1000 per well. After 3 days co-culture, the young APC were harvested for analysis.

**Lentivirus-mediated knockdown and overexpression experiments.**

Lentiviruses were produced as previously described^20^. For knockdown experiments, 293T cells were transfected with pTSB vectors carrying shRNA against *PLAU*, as well as the packaging plasmids pMD2.G and psPAX2. The no-load pTSB vectors were used as negative control, and the PLAU-shRNA sequences used in this study were listed in Supplementary Table S7. For overexpression experiments, 293T cells were transfected with pLVTH vectors carrying cDNA of *PLAU*, as well as the packaging plasmids pMD2.G and psPAX2. Lentiviral particles were collected at 24 hours and 72 hours after transfection and concentrated by ultracentrifugation. Concentrated viruses were used for cell transduction.

The silencing and overexpressing efficiency were quantified by these equations:

*PLAU* silencing efficiency = (mean relative expression of shNC - relative expression of sh*PLAU*)/mean relative expression of shNC*100%

*PLAU* overexpressing efficiency = relative expression of pLVTH*PLAU*/mean relative expression of pLVTHNC*100%

**Quantitative real-time PCR (qRT-PCR)**

Quantitative real-time PCR was performed as previously described^21^. RNA was isolated using RNAiso Plus (TakaRa). cDNA was synthesized from 2μg of RNA with ReverTra Ace qPCR RT Master Mix (TOYOBO). RT-PCR was performed using SYBR Green QPCR Master Mix (TakaRa) on a Light Cycler apparatus (Roche 480II). Values were calculated with the 2-△△Ct method and normalized to *ACTB* expression. All primers used in this study are summarized in Supplementary Table S7.

**Adipogenic differentiation and quantification**

Adipogenic differentiation was performed as previously described^17^. APC were plated at 5🞨10^4^ cells/well in 24 well-plates (Costar). After adhesion, the cells were treated with adipogenic induction medium (high glucose DMEM, 10% FBS, 0.5 mM 3-Isobutyl-1-methylxanthine, 0.5 mM hydrocortisone, 60 μM indomethacin) for 14 days, the medium were changed every two days. The cells were fixed with 4% formaldehyde before staining with Oil Red (Sigma#O0625). For quantification, the Oil Red staining was eluated with isopropanol, the absorbance was read at 510 nm by microplate reader (Tecan).

**Senescence-associated β-galactosidase (SA-β-gal) staining**

SA-β-gal staining was performed as manufacturer’s recommendations (Beyotime#C0602). Briefly, cells were washed in PBS, fixed in SA-β-gal staining stationary liquid at room temperature for 15 min, and stained in freshly prepared staining solution at 37°C overnight. The nuclear DNA was stained with DAPI. Average optical density of SA-β-gal staining is analyzed by Image J.

**Statistical analysis**

Data are presented as the mean ± s.d.. Cell type proportions analysis was performed by propeller and moderated t-test. Other statistical analysis was performed using GraphPad Prism with unpaired two-tailed Student’s t-test if not specifically stated. *P values* lower than 0.05 are considered statistically significant. *, **, *** and **** indicate *P* < 0.05, *P* < 0.01, *P* < 0.001 and *P* < 0.0001, respectively.

**Extended Discussion**

Adipose tissues distribute throughout the body, different adipose depots differ a lot in their characteristics and functions^22^. Subcutaneous adipose tissue (SAT) and visceral adipose tissue (VAT) have almost opposite characteristics and are often used for comparative analysis. While increased VAT mass is associated with metabolic dysfunction, increased SAT is protective^23^. Although in this study we only focused on the aging-dependent changes of SAT, the aging of VAT at single cell level is also worth exploring. Similar to subcutaneous adipose tissue (SAT), the aging process of mice visceral adipose tissue (VAT) is also accompanied by the emergency of inflammatory macrophages^24^. Besides, the differentiation potential of human omental preadipocytes was completely inhibited by conditioned medium of THP1-macrophage^25^, which means preadipocytes in VAT are also influenced by macrophages. However, several studies have pointed out that VAT and SAT exhibit different aging response pattern^26-28^, suggesting that the features found in SAT might not always be consistent in VAT. Based on the findings above, we believe that macrophages are involved in the regulation of VAT aging process. However, a series of studies are needed to determine the phenotypic alterations of macrophages, the specific APC populations regulated by macrophages, as well as the underlying mechanisms during VAT aging, which could be our future directions.

In this study, we identified an aging-related dysfunctional and inflammatory PLAU^+^ APC population*.* The low expression level of functional gene *CFD* means the impaired adipogenic differentiation capacity of PLAU^+^ APC population. The aging-dependent accumulation of PLAU^+^ APC suggests that its dysfunctional property might contribute to reduced lipid storage capacity, ectopic fat accumulation, and finally metabolic diseases caused by aging^29^. On the other hand, the high expression level of chemokine genes of PLAU^+^ APC reveals its inflammatory property. Although the inflammatory property of aged APC has been well documented^30^, studies at the cell subpopulation level have been lacking. Our research fills a gap in this field, suggesting that the inflammatory PLAU^+^ APC might be a specific cell source of aging-related adipose tissue inflammation.

*PLAU* encodes urokinase-type plasminogen activator, a secreted serine protease that converts plasminogen to plasmin, participating in fibrin degradation and ECM remodeling. Our GO analysis of APC also highlights the changes in ECM organization during aging. Previous researches indicated that ECM remodeling has a great effect on obesity and obesity induced inflammation and insulin resistance^31-33^, while less was concerned about the effect on aging. Thus, combine with our results, the dysfunctional and inflammatory PLAU^+^ APC may play a role in adipose ECM remodeling during aging, and it may be a direction for future research on adipose tissue aging.

In conclusion, the discovery of this novel aging-dependent APC population in human SAT and its marker gene *PLAU* provide potential diagnostic basis and therapeutic target for aging-related adipose tissue dysfunction. Future researches could targeted deliver anti-inflammatory and adipogenesis-promoting drugs to rejuvenate this dysfunctional and inflammatory APC population; or targeted ablate this dysfunctional and inflammatory APC population to block aging-related adipose tissue inflammation. Such strategies might be more effective in preventing or treating metabolic diseases caused by aging-related adipose tissue dysfunction.

**Reference**

1. Traktuev, D.O.*, et al.* A population of multipotent CD34-positive adipose stromal cells share pericyte and mesenchymal surface markers, reside in a periendothelial location, and stabilize endothelial networks. *Circ Res* **102**, 77-85 (2008).

2. Ren, X.*, et al.* COVID-19 immune features revealed by a large-scale single-cell transcriptome atlas. *Cell* **184**, 1895-1913 e1819 (2021).

3. Yin, H.*, et al.* RNA m6A methylation orchestrates cancer growth and metastasis via macrophage reprogramming. *Nat Commun* **12**, 1394 (2021).

4. Zheng, G.X.*, et al.* Massively parallel digital transcriptional profiling of single cells. *Nat Commun* **8**, 14049 (2017).

5. Heaton, H.*, et al.* Souporcell: robust clustering of single-cell RNA-seq data by genotype without reference genotypes. *Nat Methods* **17**, 615-620 (2020).

6. Wu, S.J.*, et al.* Single-cell CUT&Tag analysis of chromatin modifications in differentiation and tumor progression. *Nat Biotechnol* (2021).

7. Butler, A., Hoffman, P., Smibert, P., Papalexi, E. & Satija, R. Integrating single-cell transcriptomic data across different conditions, technologies, and species. *Nat Biotechnol* **36**, 411-420 (2018).

8. Yu, G., Wang, L.G., Han, Y. & He, Q.Y. clusterProfiler: an R package for comparing biological themes among gene clusters. *OMICS* **16**, 284-287 (2012).

9. Phipson, B.*, et al.* Propeller: testing for differences in cell type proportions in single cell data. *Bioinformatics* (2022).

10. Wang, Y.*, et al.* iTALK: an R Package to Characterize and Illustrate Intercellular Communication. *bioRxiv* (2019).

11. Vento-Tormo, R.*, et al.* Single-cell reconstruction of the early maternal-fetal interface in humans. *Nature* **563**, 347-353 (2018).

12. Gu, Z., Gu, L., Eils, R., Schlesner, M. & Brors, B. circlize Implements and enhances circular visualization in R. *Bioinformatics* **30**, 2811-2812 (2014).

13. La Manno, G.*, et al.* RNA velocity of single cells. *Nature* **560**, 494-498 (2018).

14. Bergen, V., Lange, M., Peidli, S., Wolf, F.A. & Theis, F.J. Generalizing RNA velocity to transient cell states through dynamical modeling. *Nat Biotechnol* **38**, 1408-1414 (2020).

15. Cao, J.*, et al.* The single-cell transcriptional landscape of mammalian organogenesis. *Nature* **566**, 496-502 (2019).

16. Dai, J.*, et al.* Kdm6b regulates cartilage development and homeostasis through anabolic metabolism. *Ann Rheum Dis* **76**, 1295-1303 (2017).

17. Zhou, W.*, et al.* Single-Cell Profiles and Clinically Useful Properties of Human Mesenchymal Stem Cells of Adipose and Bone Marrow Origin. *Am J Sports Med* **47**, 1722-1733 (2019).

18. in *The Impact of Food Bioactives on Health: in vitro and ex vivo models* (eds. Verhoeckx, K.*, et al.*) (Cham (CH), 2015).

19. Baar, M.P.*, et al.* Targeted Apoptosis of Senescent Cells Restores Tissue Homeostasis in Response to Chemotoxicity and Aging. *Cell* **169**, 132-147 e116 (2017).

20. Zou, Z.*, et al.* A Single-Cell Transcriptomic Atlas of Human Skin Aging. *Dev Cell* **56**, 383-397 e388 (2021).

21. Hindson, C.M.*, et al.* Absolute quantification by droplet digital PCR versus analog real-time PCR. *Nat Methods* **10**, 1003-1005 (2013).

22. Zwick, R.K., Guerrero-Juarez, C.F., Horsley, V. & Plikus, M.V. Anatomical, Physiological, and Functional Diversity of Adipose Tissue. *Cell Metab* **27**, 68-83 (2018).

23. Tran, T.T., Yamamoto, Y., Gesta, S. & Kahn, C.R. Beneficial effects of subcutaneous fat transplantation on metabolism. *Cell Metab* **7**, 410-420 (2008).

24. Lumeng, C.N.*, et al.* Aging is associated with an increase in T cells and inflammatory macrophages in visceral adipose tissue. *J Immunol* **187**, 6208-6216 (2011).

25. Constant, V.A., Gagnon, A., Landry, A. & Sorisky, A. Macrophage-conditioned medium inhibits the differentiation of 3T3-L1 and human abdominal preadipocytes. *Diabetologia* **49**, 1402-1411 (2006).

26. Buffolo, M.*, et al.* Identification of a Paracrine Signaling Mechanism Linking CD34(high) Progenitors to the Regulation of Visceral Fat Expansion and Remodeling. *Cell Rep* **29**, 270-282 e275 (2019).

27. Van Harmelen, V., Rohrig, K. & Hauner, H. Comparison of proliferation and differentiation capacity of human adipocyte precursor cells from the omental and subcutaneous adipose tissue depot of obese subjects. *Metabolism* **53**, 632-637 (2004).

28. Nguyen, H.P.*, et al.* Aging-dependent regulatory cells emerge in subcutaneous fat to inhibit adipogenesis. *Dev Cell* **56**, 1437-1451 e1433 (2021).

29. Gustafson, B., Hedjazifar, S., Gogg, S., Hammarstedt, A. & Smith, U. Insulin resistance and impaired adipogenesis. *Trends Endocrinol Metab* **26**, 193-200 (2015).

30. Tchkonia, T.*, et al.* Fat tissue, aging, and cellular senescence. *Aging Cell* **9**, 667-684 (2010).

31. Unamuno, X.*, et al.* NLRP3 inflammasome blockade reduces adipose tissue inflammation and extracellular matrix remodeling. *Cell Mol Immunol* **18**, 1045-1057 (2021).

32. Ruiz-Ojeda, F.J., Mendez-Gutierrez, A., Aguilera, C.M. & Plaza-Diaz, J. Extracellular Matrix Remodeling of Adipose Tissue in Obesity and Metabolic Diseases. *Int J Mol Sci* **20**(2019).

33. Vaittinen, M.*, et al.* MFAP5 is related to obesity-associated adipose tissue and extracellular matrix remodeling and inflammation. *Obesity (Silver Spring)* **23**, 1371-1378 (2015).

**
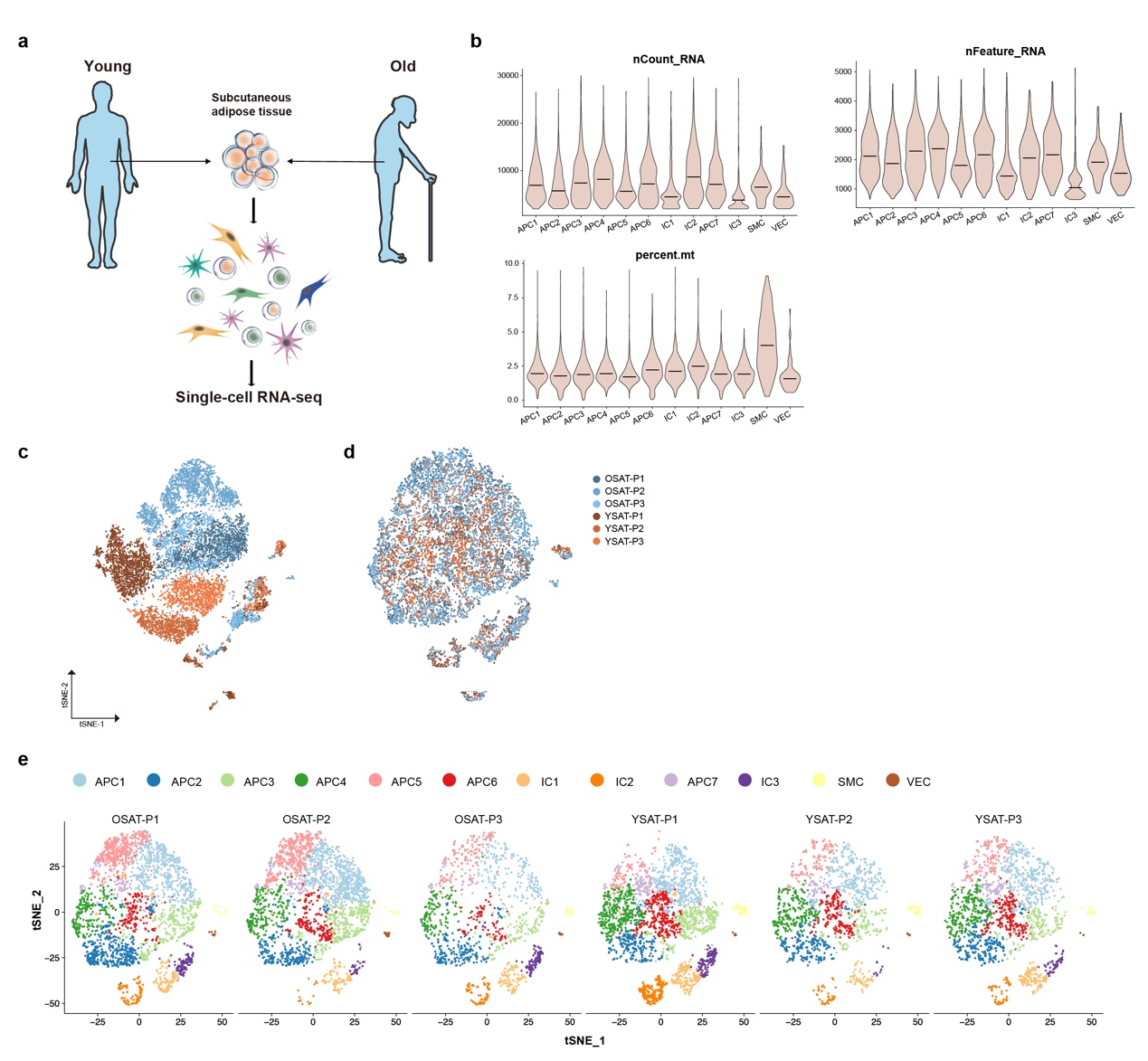
Supplementary Fig. S1. Quality control and integrated analysis on human SAT cells derived from different individuals. a,** Schematic diagram of the experimental workflow, samples from 3 young individuals and 3 old individuals. **b,** Violin plot of number of counts (nCount_RNA), number of unique genes (nFeature_RNA), percentage of mitochondrial gene (percent.mt) across human SAT cells from 3 young and 3 old individuals. **c,** t-SNE plot shows the distribution of each individual before integration processing. **d,** t-SNE plot shows the distribution of each individual after integration processing. **e,** t-SNE visualization of the clusters in each individual. YSAT, young subcutaneous adipose tissues; OSAT, old subcutaneous adipose tissues.

**
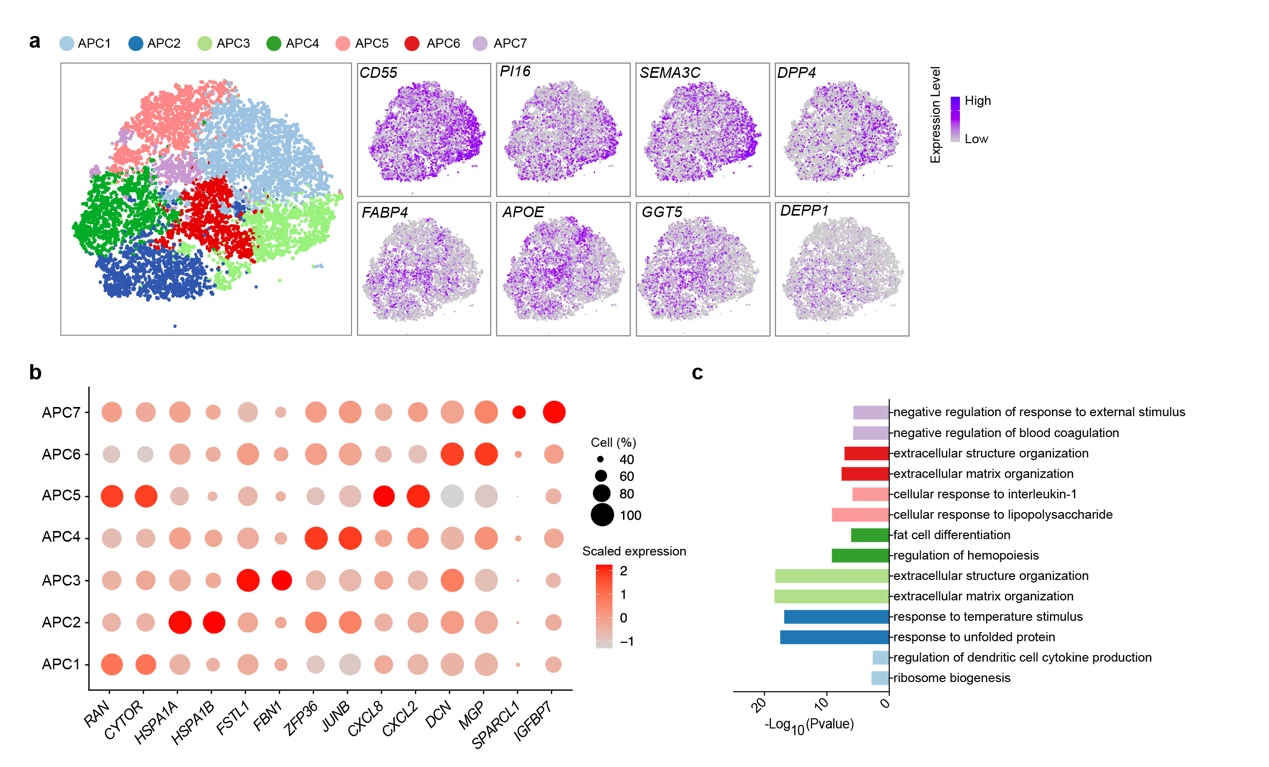
Supplementary Fig. S2. Transcriptional classification identified seven subpopulations of human APC. a,** t-SNE plots show the APC subpopulations and the expression distribution of stem cell marker genes (*CD55*, *PI16*, *SEMA3C*, *DPP4*) and adipogenesis-related marker genes (*FABP4*, *APOE*, *GGT5*, *DEPP1*). Left, cells colored by cell type. Right, the color key from gray to purple indicates low to high gene expression levels. **b,** Dot plot shows the expression levels of representative cell-type-specific marker genes across all these 7 APC subpopulations. The node size positively correlates with the percentage of the cells within a subpopulation positive for a given marker. The color encodes the scaled average expression levels of feature genes across all cells within a subpopulation. **c,** Representative enriched GO terms for each APC cell type.

**
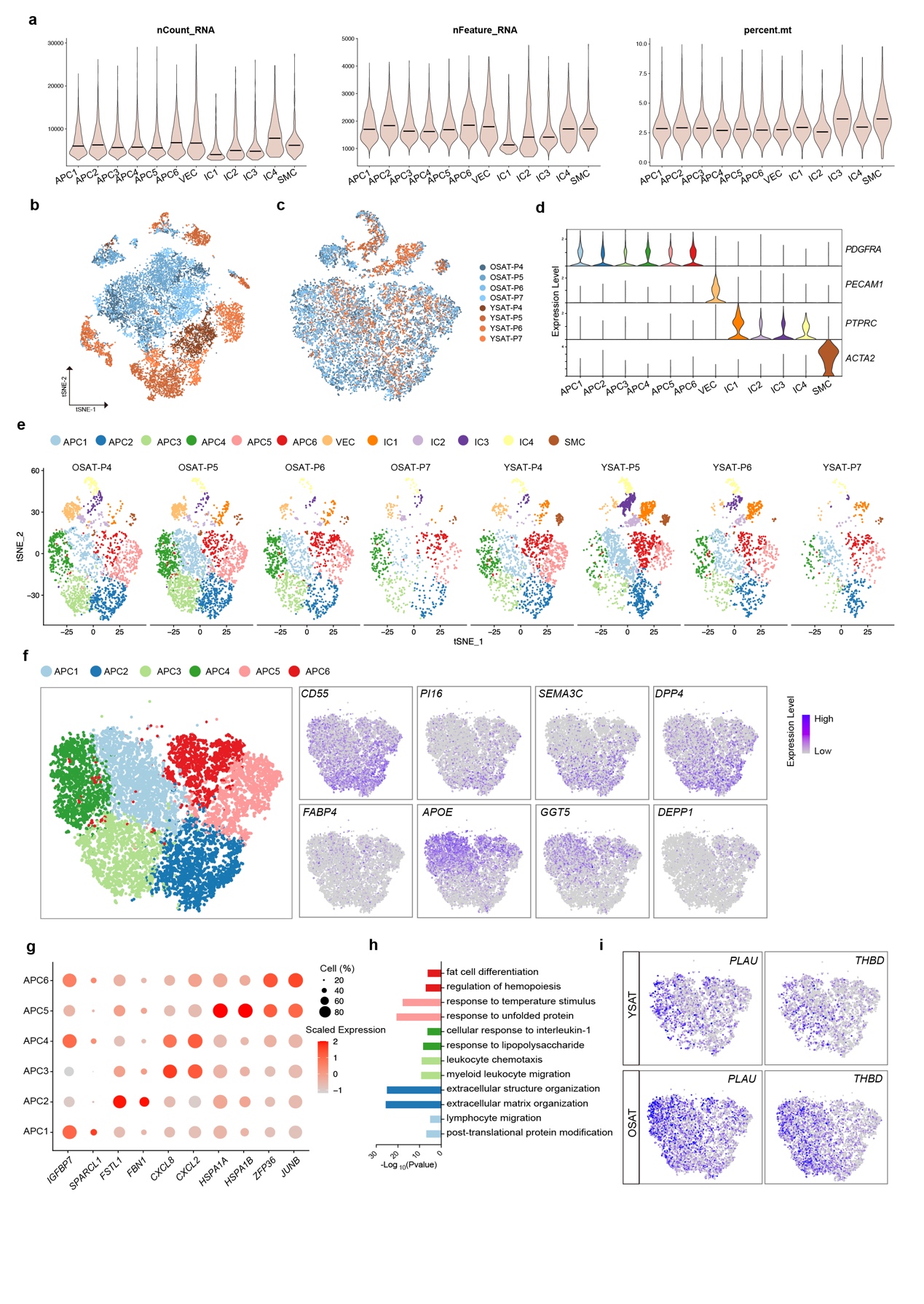
Supplementary Fig. S3. scRNA-seq on human SAT cells from another cohort verified the accumulation of the dysfunctional PLAU^+^ APC during aging. a,** Violin plot of number of counts (nCount_RNA; left), number of unique genes (nFeature_RNA; middle), percentage of mitochondrial gene (percent.mt; right) across human SAT cells from 4 young and 4 old individuals. **b,** t-SNE plot shows the distribution of each individual before integration processing. **c,** t-SNE plot shows the distribution of each individual after integration processing. **d,** Violin plots show the linage marker genes expression level in all cell clusters. **e,** t-SNE visualization of the clusters in each individual. **f,** t-SNE plots showing the APC subpopulations and the expression distribution of stem cell marker genes (*CD55*, *PI16*, *SEMA3C*, *DPP4*) and adipogenesis-related marker genes (*FABP4*, *APOE*, *GGT5*, *DEPP1*). Left, cells colored by cell type. Right, the color key from gray to purple indicates low to high gene expression levels. **g,** Dot plot shows the expression levels of representative cell-type-specific marker genes across all these 6 APC subpopulations. **h,** Representative enriched GO terms for each APC cell type. **i,** t-SNE plots show the expression level of *THBD* and *PLAU* in all APC clusters in young and old individuals. YSAT, young subcutaneous adipose tissues; OSAT, old subcutaneous adipose tissues.

**
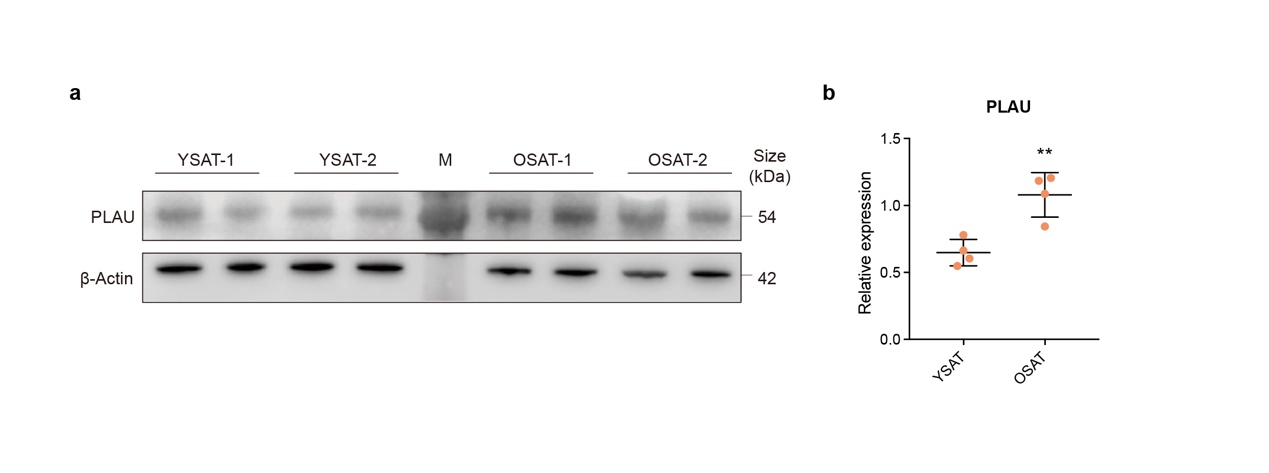
Supplementary Fig. S4. Western blotting shows SAT of old individuals express higher PLAU protein level. a,** Western blotting images. **b,** Quantitative analysis of PLAU protein expression level. YSAT, young subcutaneous adipose tissues; OSAT, old subcutaneous adipose tissues; M, protein marker.

**
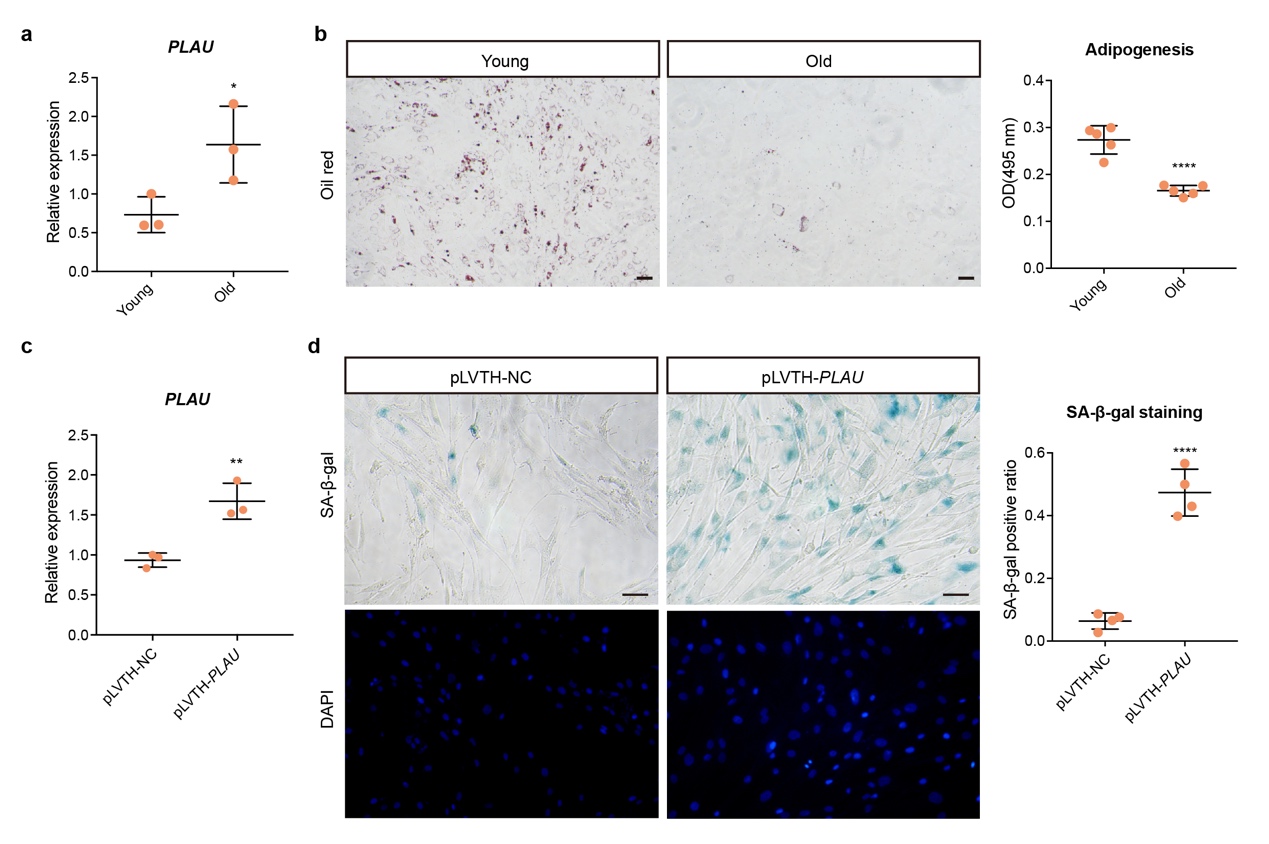
Supplementary Fig. S5. *PLAU* overexpression induces APC senescence. a,** qRT-PCR shows *PLAU* expression level of human APC derived from young and old individuals. Data are presented as the mean ± s.d. of 3 biological replications. **b,** Representative images of Oil Red staining show the adipogenic differentiation capacity of human APC derived from young and old individuals. Data are presented as the mean ± s.d. of 4 biological replications. **c,** qRT-PCR shows *PLAU* expression level of human APC after *PLAU* overexpression. Data are presented as the mean ± s.d. of 4 biological replications. **d,** Representative images of SA-β-gal staining and DAPI staining of the same view of human APC upon overexpression of *PLAU*. Scale bar, 50μm. Data are presented as the mean ± s.d. of 4 fields. **P* < 0.05. ***P* < 0.01. *****P* < 0.0001.

**
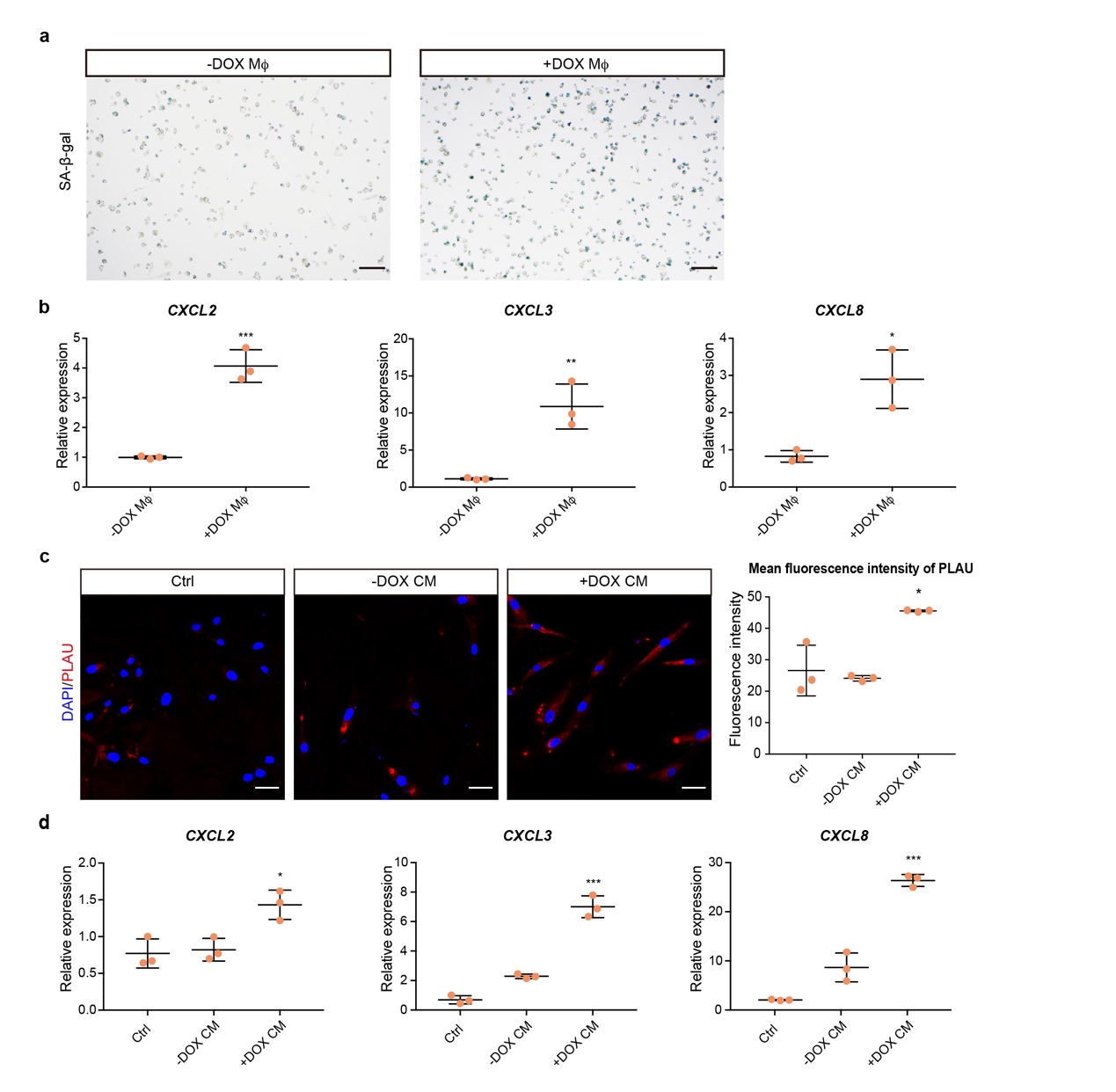
Supplementary Fig. S6. DOX induced senescent macrophage cell line. a,** Representative images of SA-β-gal staining of DOX treated macrophages. **b,** qRT-PCR shows the expression levels of *CXCL2*, *CXCL3*, *CXCL8* of DOX treated macrophages. Data are presented as the mean ± s.d. of 3 batches of macrophages. **c,** Representative images of immunofluorescence staining of PLAU in conditioned medium treated APC. Data are presented as the mean ± s.d. of 3 fields. **d**, qRT-PCR shows the expression levels of *CXCL2*, *CXCL3*, *CXCL8* of APC treated by senescent macrophage conditioned medium. Data are presented as the mean ± s.d. of APC derived from 3 young individuals. -DOX MΦ, macrophages without DOX treatment; +DOX MΦ, macrophages treated by DOX; -DOX CM, conditioned medium of macrophages without DOX treatment; +DOX CM, conditioned medium of macrophages treated by DOX. **P* < 0.05, ***P*< 0.01, ****P* < 0.001.

**
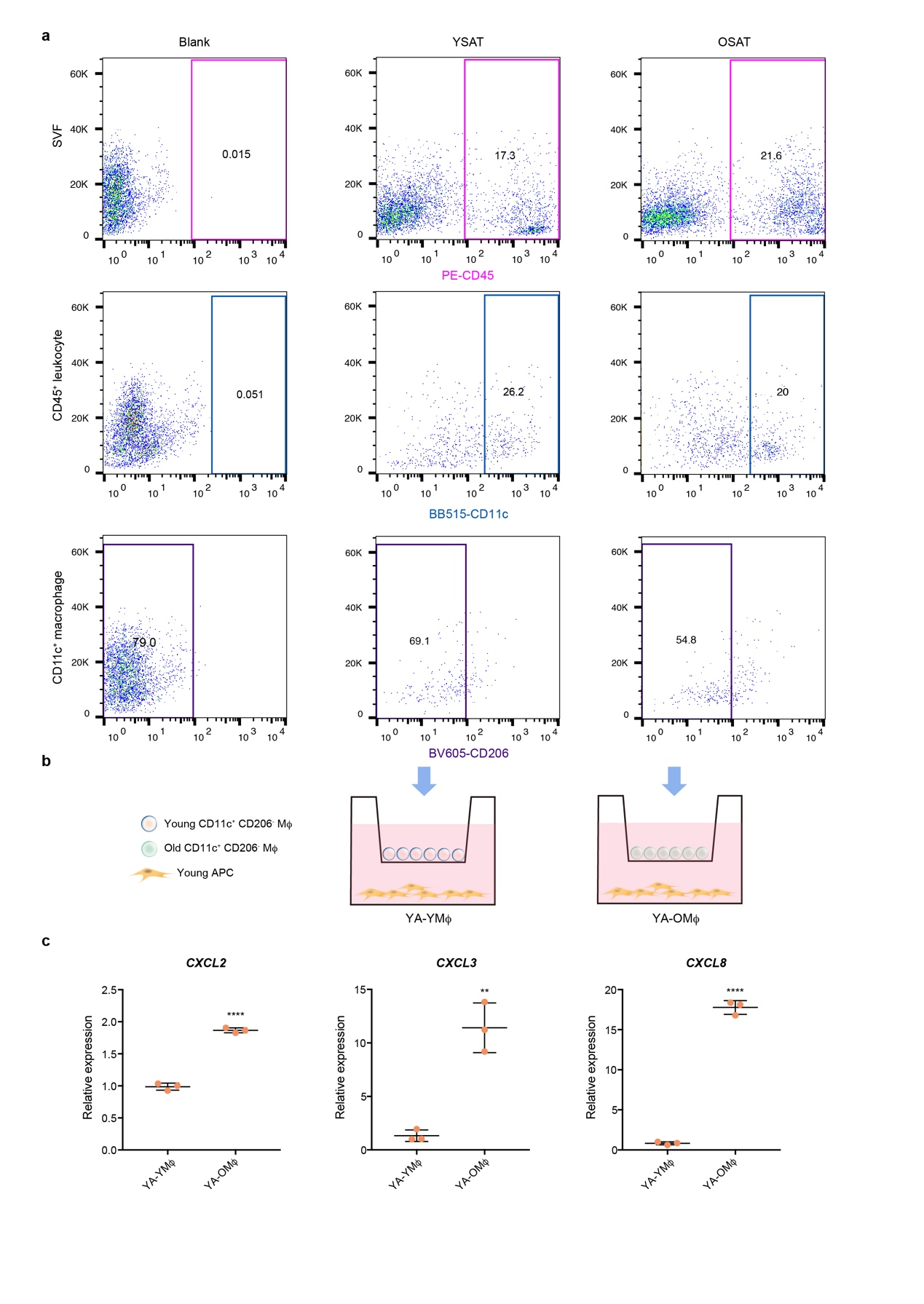
Supplementary Fig. S7. Macrophages isolated from SAT of old individual promote the inflammatory phenotype of young APC. a,** Flow cytometry gating strategy for isolating CD11c^+^CD206^-^ macrophages from SAT of young and old individuals. **b,** Schematic diagram of CD11c^+^CD206^-^ macrophages and APC co-culture system. **c,** qRT-PCR shows the expression levels of *CXCL2*, *CXCL3*, *CXCL8* of young APC after 3 days co-culture with isolated macrophages. Data are presented as the mean ± s.d. of three technical repetitions. YSAT, young subcutaneous adipose tissues; OSAT, old subcutaneous adipose tissues; YA-YMΦ, young APC and young macrophages co-culture system; YA-OMΦ, young APC and old macrophages co-culture system. ***P* < 0.01. *****P* < 0.0001.

**Original film of Western blot**

**
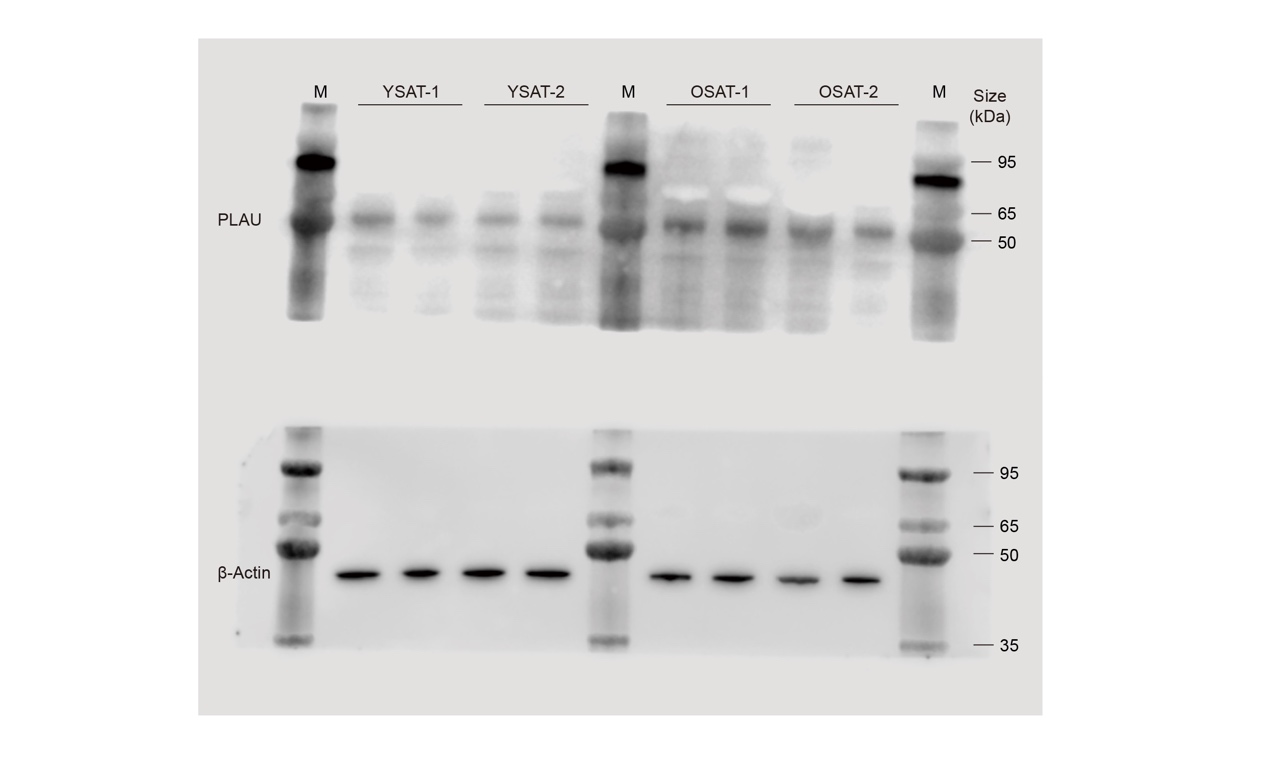
**

**Supplementary Table S1.** Information of the human adipose samples in scRNA-seq.

**Supplementary Table S2.** GO analysis results of conserved marker genes of each APC cluster of the first cohort.

**Supplementary Table S3.** Aging-dependent upregulated top 50 gene list of APC5.

**Supplementary Table S4.** GO analysis results of conserved marker genes of each APC cluster of the second cohort.

**Supplementary Table S5.** Conserved marker genes of each ICS cluster of the first cohort.

**Supplementary Table S6.** Characteristics of scRNA-seq data quality.

**Supplementary Table S7.** Primer sequences and *PLAU*-shRNA sequences.
